# Differences in susceptibility to German cockroach frass and its associated proteases in induced allergic inflammation in mice

**DOI:** 10.1186/1465-9921-8-91

**Published:** 2007-12-08

**Authors:** Kristen Page, Kristin M Lierl, Nancy Herman, Marsha Wills-Karp

**Affiliations:** 1 Division of Critical Care Medicine, Cincinnati Children's Hospital Medical Center, Cincinnati, OH, USA; 2 Division of Immunobiology, Cincinnati Children's Hospital Medical Center, Cincinnati, OH, USA; 3 Department of Pediatrics, University of Cincinnati, Cincinnati, Ohio, USA

## Abstract

**Background:**

Cockroach exposure is a major risk factor for the development of asthma. Inhalation of fecal remnants (frass) is the likely sensitizing agent; however isolated frass has not been tested for its ability to induce experimental asthma in mice.

**Methods:**

Mice (Balb/c or C57Bl/6) were sensitized and challenged with GC frass or GC frass devoid of proteases and measurements of airway inflammation and hyperresponsiveness were performed (interleukin (IL)-5, -13, and interferon gamma (IFNγ) levels in bronchoalveolar lavage fluid, serum IgE levels, airway hyperresponsiveness, cellular infiltration, and mucin production).

**Results:**

Sensitization and challenge of Balb/c mice with GC frass resulted in increased airway inflammation and hyperresponsiveness. C57Bl/6 mice were not susceptible to this model of sensitization; however they were sensitized to GC frass using a more aggressive sensitization and challenge protocol. In mice that were sensitized by inhalation, the active serine proteases in GC frass played a role in airway hyperresponsiveness as these mice had less airway hyperresponsiveness to acetylcholine and less mucin production. Proteases did not play a role in mediating the allergic inflammation in mice sensitized via intraperitoneal injection.

**Conclusion:**

While both strains of mice were able to induce experimental asthma following GC frass sensitization and challenge, the active serine proteases in GC frass only play a role in airway hyperresponsiveness in Balb/c mice that were susceptible to sensitization via inhalation. The differences in the method of sensitization suggest genetic differences between strains of mice.

## Introduction

The principal domestic cockroach species that commonly infests homes in the United States are the German cockroaches (GC; *Blattella germanica*). During infestation, cockroaches (CR) produce a variety of substances that may be allergenic including exoskeleton, secretions, egg castings and fecal remnants (frass). Of these, the whole body CR and frass have been shown to contain significant and similar allergenic activity [1], suggesting that most of the allergenic activity is released in the frass. Although the sensitization route of CR exposure is not fully understood, it is likely that inhalation of frass is a main route of exposure. Frass particles are very dry; therefore they may incorporate into house dust more readily than the hard chitinous materials. In fact, significant quantities of CR antigen were found in household dust [2,3]. While frass contains high levels of the cockroach allergens Bla g1 and Bla g2 [4], it also contains active serine proteases [5,6], coliforms [7], pheromones, and a number of proteins and other components. While frass is the most likely source of GC allergen exposure, isolated GC frass has never been used as a sensitizing agent to induce the experimental asthma phenotype in mice.

A number of studies have strongly suggested that cockroach allergens are a significant cause of asthma (for review [8]) and that it may be more important relative to exposure to other allergens. Indoor concentrations of CR allergen, but not house dust mite, were found to be significantly associated with recurrent wheezing and asthma [9]. For example, one study showed that in 63 children less than 4 years of age, 24% were sensitized to cockroach allergen [10]. In inner-city asthmatics which require frequent emergency room and hospital visits, sensitization to cockroach allergen is highly prevalent, suggesting the likelihood that cockroach exposure may be responsible for inducing their symptoms [11-14]. Early life cockroach allergen exposure was shown to predict allergen-specific responses by 2 years of age [15]. A correlation in the rise of adolescent asthma in densely populated areas and allergies to cockroach antigen have been shown [16,17]. While this increase cannot be solely linked to cockroach exposure, roughly 60% of inner city children have highly elevated IgE levels specific for cockroach [18]. Together these studies have led investigators to speculate that cockroach allergens are important mediators of allergy and asthma and therefore warrant their further study.

Much of the work in murine models of allergen-induced allergic inflammation has been performed using ovalbumin (OVA) as a sensitizing agent. In order to elicit an allergic response to OVA, mice must be immunized by intraperitoneal injection of OVA bound to an adjuvant such as aluminum hydroxide (alum). While allergen-induced allergic inflammation is detected, these studies do not mirror human susceptibility of this disease. Therefore in this report we attempt to address not only the use of GC frass as a sensitizing agent, but also to demonstrate a model of allergic sensitization in mice that mirrors the human etiology of allergic asthma. We will use two methods of sensitization to confirm the role of GC frass in mediating allergen-induced allergic inflammation in mice. The first method is sensitization and challenge by intratracheal inhalation, and the second method is sensitization by intraperitoneal injection with GC frass bound to alum with an intratracheal challenge. In addition, since GC frass contains active serine proteases [6] we will investigate the role of active proteases in regulating airway inflammation and airway hyperresponsiveness.

## Materials and methods

### Cockroach frass

Fecal remnants (frass) were collected from German cockroaches (*Blattella germanica*) and reconstituted as previously described [5]. The frass preparation was frozen in aliquots and used throughout the entire experiment. To inhibit protease activity in frass, frass was pre-treated with aprotinin (a specific inhibitor of serine proteases; 10 μg/ml for 30 min at 37°C) prior to use. Protease activity was determined using the Azocoll assay as previously described [19]. GC frass was determined to contain 19 μg protease activity/mg frass and aprotinin treatment inhibited 80% of the protease activity [6] and will hence be referred to as protease-free GC frass. Endotoxin levels were determined by Limulus Amebocyte assay by Charles Rivers Laboratories (Charleston, SC) to be 922.93 ng endotoxin/mg frass. Bla g2 levels were measured by ELISA (Indoor Biotechnologies, Charlottesville, VA) according to manufacturers' specifications and determined to be 5.3 μg/mg frass.

### Animals

Six week old female Balb/c or C57Bl/6 mice were obtained from Jackson Laboratory (Bar Harbor, ME) and housed in a laminar hood in a virus-free animal facility. These studies conformed to the principles for laboratory animal research outlined by the Animal Welfare Act and the Department of Health, Education, and Welfare (National Institutes of Health). These studies were approved by the Cincinnati Children's Hospital Medical Center Institutional Animal Care and Use Committee.

### Sensitization and challenge protocols

**Figure 1:**
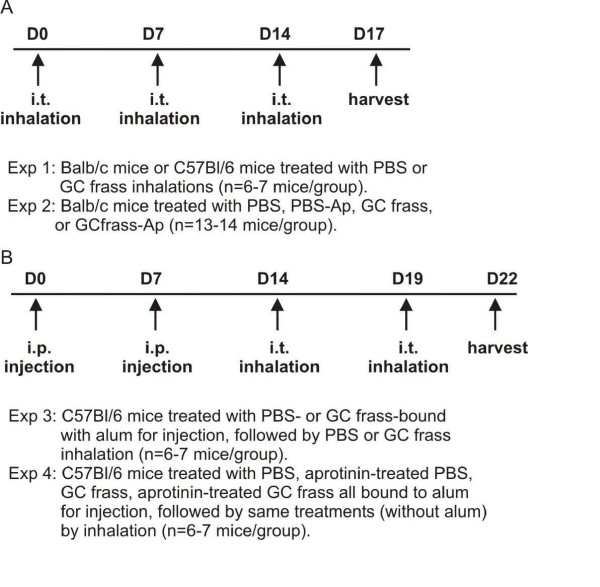
Sensitization and challenge protocols. A. Protocol for Balb/c mice. B. Protocol for C57Bl/6 mice.

Murine strains are known to exhibit different immune responses, with Balb/c mice being more responsive and C57Bl/6 mice being less responsive to allergen challenge. Therefore, we compared a sensitization by inhalation only protocol to the standard sensitization by intraperitoneal injection followed by inhalation challenge. In one method, GC frass was delivered via intratracheal aspiration challenge. Briefly, anesthetized mice (45 mg/kg ketamine and 8 mg/kg xylazine) were suspended on a 60 degree incline board. With the tongue gently extended, a 40 μl aliquot of PBS or GC frass is placed in the back of the oral cavity and aspirated by the mouse [20]. Balb/c mice were given three challenges of PBS (40 μl) or GC frass (40 μg/40 μl) on days 0, 7, and 14 and harvested on day 17 (Figure 1A). In some experiments, mice were also treated with PBS pretreated with aprotinin (10 μg/ml) or GC frass pretreated with aprotinin. In the other method, mice were immunized with PBS or 10 μg/ml GC frass bound to alum (Imject Alum; Pierce Biotechnology, Rockford, IL) on day 0 and 7, followed by intratracheal inhalation challenges with GC frass (40 μg/40 μl) on days 14 and 19. Mice were harvested on day 22 (Figure 1B). In some experiments, mice were sensitized and challenged with aprotinin-treated PBS or GC frass.

### Airway hyperresponsiveness measurements

Allergen-induced AHR was determined as we have previously described [21]. Briefly, mice were anesthetized 72 hours after the last GC frass exposure, intubated and ventilated at a rate of 120 breaths per minute with a constant tidal volume of air (0.2 ml), and paralyzed with decamethonium bromide (25 mg/kg). After establishment of a stable airway pressure, 25 μg/kg weight of acetylcholine was injected i.v. and dynamic airway pressure (airway pressure time index [APTI] in cm-H_2_O × sec^-1^) was followed for 5 minutes.

### Assessment of airway inflammation

Lungs were lavaged thoroughly with 1 ml of Hanks balanced salt solution without calcium or magnesium. The lavage fluid was centrifuged (1,800 rpm for 10 min), the supernatant was removed for cytokine analysis and immediately stored at -80°C. Total cell numbers were counted on a hemocytometer. Smears of BAL cells prepared with a Cytospin II (Shandon Thermo, Waltham, MA) were stained with Diff-Quick (Thermo Electron Corporation, Pittsburg, PA) solution for differential cell counting.

### Cytokine production

Liberase/DNase I digests of the lung were prepared to obtain single lung cell suspensions. Single cell suspensions (2.5 × 10^5^) were incubated for 72 hours in culture medium (RPMI) or in RPMI treated with Conconavalin A (10 μg/ml) and supernatants were analyzed by ELISA for TH2 cytokine (IL-5 and IL-13) or TH1 cytokine (interferon (IFN) γ) expression as previously described [22].

### Histology

Whole lungs were removed and formalin fixed. Lungs were embedded in paraffin, sectioned, and stained with haematoxylin and eosin (H&E) and Periodic Acid Schiff (PAS). To quantify mucin production, we counted airways and determined the percentage of mucin stained airways (mean ± SEM; n= 3 slides per condition). Next, we picked representative airways and counted total and mucin positive cells in that airway and determined the percentage of mucin positive cells (mean ± SEM; n = 5 airways per condition).

### Statistical analysis

When applicable, statistical significance was assessed by one-way analysis of variance (ANOVA). Differences identified by ANOVA were pinpointed by Student-Newman-Keuls' multiple range test.

## Results

### GC frass induced airway inflammation and hyperresponsiveness in mice

**Figure 2:**
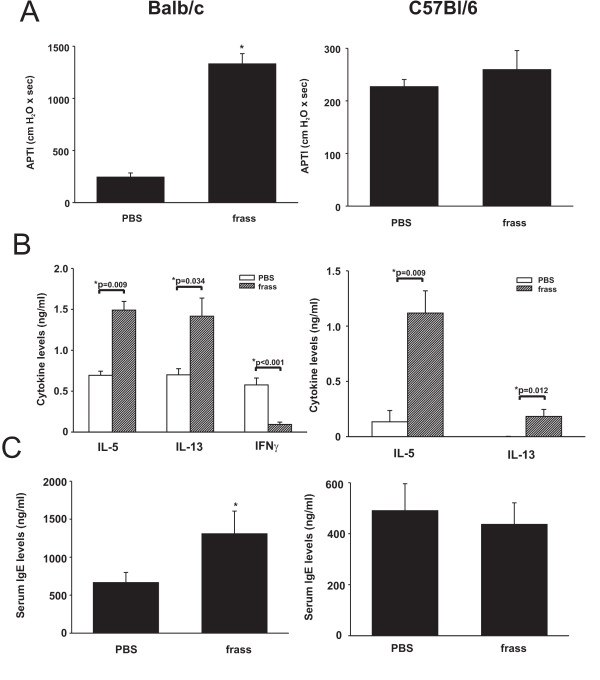
GC frass-induced experimental allergic asthma in Balb/c mice. Balb/c mice were challenged by intratracheal inhalation on day 0, 7, and 14 with PBS (40 μl) or GC frass (40 μg/40 μl). On day 17, mice were anesthetized and acetylcholine was injected after establishment of a stable airway pressure. A. AHR was measured as airway pressure time index (APTI) in cm-H_2_O × sec ^-1 ^(* p < 0.001). B. Lungs from the mice were excised; cells dissociated and maintained in a single suspension culture for 3 days in the presence of Con A (10 μg/ml). Supernatants were removed and ELISAs were run for IL-5, IL-13 and IFNγ. These data are represented as cytokine (listed on the x-axis) in ng/ml from PBS or frass treated mice. C. Serum IgE levels were analyzed by ELISA (*p = 0.001). In all cases the data are expressed as mean ± SEM and represent 6–7 mice per group and statistical significance determined by ANOVA.

**Figure 3:**
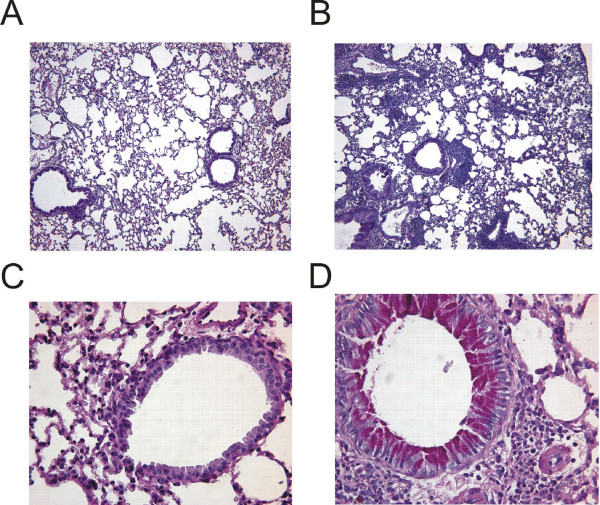
Histological assessment of lung sections from PBS- or GC frass- exposed Balb/c mice. Haematoxylin and eosin (H&E) staining of sectioned lungs from PBS (A) and GC frass (B) treated Balb/c mice. Periodic Acid Schiff (PAS) staining of sectioned lungs from PBS (C) and GC frass (D) treated Balb/c mice. Representative slides are shown of sections from 6–7 mice per group.

Mice were sensitized and challenged mice with GC frass via intratracheal inhalation as depicted in Figure 1A. Sensitization and challenge with GC frass significantly increased airway responsiveness to cholinergic agents in Balb/c mice but not C57Bl6 mice (Figure 2A). Allergen inhalation induced increases in the TH2 cytokines IL-5 and IL-13 in both strains of mice following allergen challenge (Figure 2B). In Balb/c mice, there was a decrease in the TH1 cytokine IFNγ following allergen challenge (Figure 2B). Serum IgE levels were increased in Balb/c, but not C57Bl6 mice following GC frass inhalation (Figure 2C). Cellular infiltration into the BAL fluid of Balb/c mice showed increased numbers of eosinophils, neutrophils, macrophages and lymphocytes following sensitization and challenge with GC frass (Table 1). Histological examination of the Balb/c mouse lung following GC frass treatment showed dense perivascular and peribronchiolar infiltrates (Figure 3 A+B) and abundant mucin in epithelial cells (Figure 3 C+D) compared to PBS treatment. No mucin was detected in PBS treated Balb/c mice, while 49 ± 1% of the airways stained positive for mucin in mice sensitized and challenged with GC frass. Of those stained airways, 88 ± 3% of the cells in the airway were positive for mucin. These data demonstrate that Balb/c mice are susceptible to GC frass-induced allergic inflammation and airway hyperresponsiveness following sensitization and challenge by intratracheal inhalation, while C57Bl/6 mice only had increases in TH2 cytokine levels, but no increase in IgE or airway hyperresponsiveness. These data suggests a genetic difference in susceptibility to inhaled allergen between these mouse strains.

**Table 1:** Differential cell count in BAL fluid of Balb/c mice

	**Mac**	**Epi**	**Eos**	**Neut**	**Lymph**
PBS	3.1 ± 1.2	3.2 ± 1.0	0	0	0.3 ± 0.2
frass	10.5 ± 1.5	4.1 ± 1.0	1.2 ± 0.9	1.3 ± 0.3	2.7 ± 0.3
p value	0.005	0.54	0.008	0.005	0.001

Balb/c mice were challenged on day 0, 7, and 14 with PBS or GC frass (40 μg). On day 17, BAL fluid was harvested and differential cell counts performed. These data represent 7 mice per group and are expressed as mean ± SEM cell number ×10^4^. Statistical significance between GC frass and PBS treatments were determined by ANOVA.

### The role of active serine proteases in mediating airway inflammation and airway hyperresponsiveness in Balb/c mice

**Figure 4:**
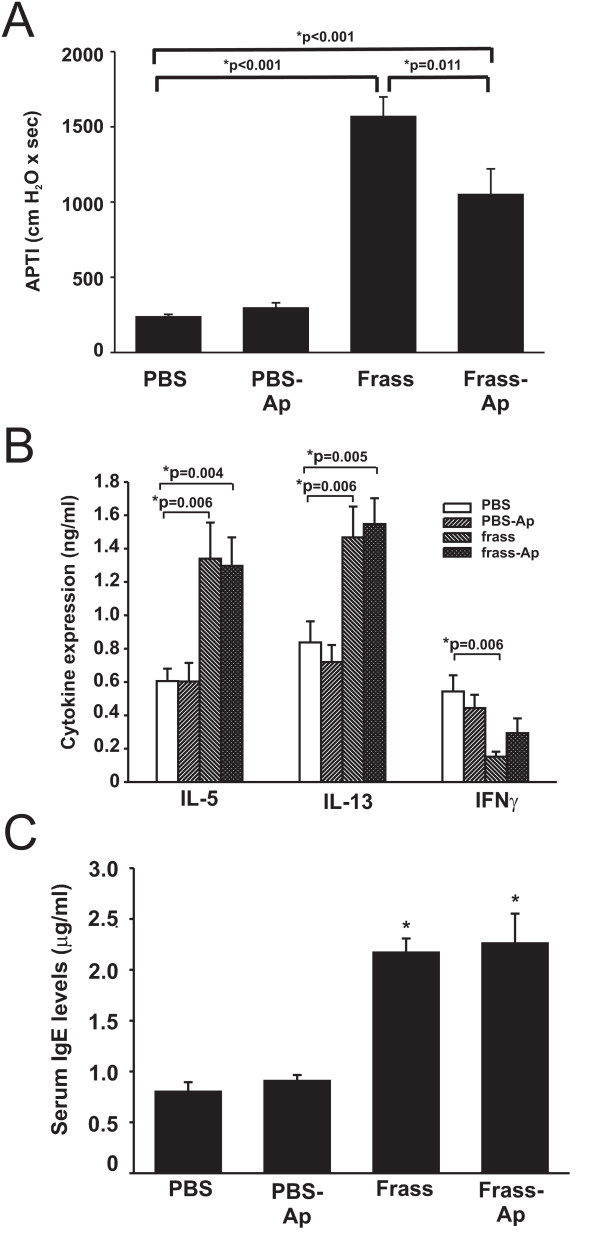
GC frass serine proteases regulate airway inflammation and airway hyperresponsiveness in Balb/c mice. Balb/c mice were challenged on day 0, 7, and 14 with PBS, PBS pretreated with aprotinin (10 μg/ml), GC frass (40 μg) or GC frass pretreated with aprotinin. On day 17, mice were anesthetized and acetylcholine was injected after establishment of a stable airway pressure. A. AHR was measured as airway pressure time index (APTI) in cm-H_2_O × sec ^-1 ^(* p < 0.001). B. Lungs from the mice were excised, and cells dissociated and maintained in a single suspension culture for 3 days in the presence of Con A (10 μg/ml). Supernatants were removed and ELISAs were run for IL-5, IL-13 and IFNγ. These data are represented as cytokine (listed on the x-axis) in ng/ml from PBS or frass treated mice. C. Serum IgE levels were analyzed by ELISA (*p < 0.001). In all cases the data are expressed as mean ± SEM and represent 13–14 mice per group and statistical significance determined by ANOVA.

**Figure 5:**
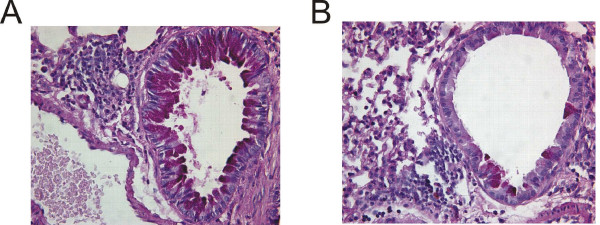
Histological assessment of lung sections from Balb/c mice exposed to GC frass or protease-depleted GC frass. Periodic Acid Schiff (PAS) staining of sectioned lungs from GC frass (A) and aprotinin-treated GC frass (D) treated Balb/c mice. Representative slides are shown of sections from 8 mice per group.

Balb/c mice were sensitized via intratracheal inhalation with PBS, aprotinin-treated PBS, GC frass, or aprotinin-treated GC frass (which we refer to as protease-free GC frass). Inhalation of protease-free GC frass resulted in reduced airway hyperresponsiveness to acetylcholine compared to protease-containing GC frass (Figure 4A). Removal of the serine proteases did not alter TH2 cytokine production, IFNγ production (Figure 4B) or serum IgE levels (Figure 4C). Aprotinin was used to inhibit serine protease activity in GC frass, which we show did not affect cytokine production, airway hyperresponsiveness or lung histology (data not shown). There was a small decrease in the amount of perivascular and peribronchiolar infiltrates in the mice challenged with aprotinin-treated GC frass than compared to GC frass as determined by H&E staining (data not shown). Notably, there was much less mucin production in mice treated with aprotinin-treated frass compared to protease containing GC frass (Figure 5 A+B). Assessment of the airways showed that GC frass inhalation resulted in positive mucin staining in 50% of the airways compared to 25% in protease-free GC frass-treated mice. Strikingly however, was the decreased amount of mucin positive cells in each airway. GC frass had 88 ± 3% mucin positive cells compared to only 22 ± 7% mucin positive cells in protease-free GC frass treated mouse airways (n = 5). Interestingly, the increase in BAL fluid eosinophils, neutrophils, macrophages and lymphocytes was unaffected by the inhibition of the serine proteases in frass (Table 2). These data suggest that GC frass derived proteases play a role in modulating airway hyperresponsiveness and mucin production, but are not required for TH2 skewing and IgE production following GC frass treatment.

**Table 2:** Differential cell count in BAL fluid of Balb/c mice treated with GC frass or protease-depleted frass

	**Mac**	**Epi**	**Eos**	**Neut**	**Lymph**
PBS	0.3 ± 0.08	0.5 ± 0.08	0	0.01 ± 0.003	0.005 ± 0.002
PBS-Ap	0.3 ± 0.09	0.3 ± 0.06	0	0.01 ± 0.002	0.005 ± 0.002
frass	1.9 ± 0.3	0.6 ± 0.1	0.4 ± 0.07	0.5 ± 0.09	0.58 ± 0.05
frass-Ap	1.6 ± 0.2	0.9 ± 0.3	0.5 ± 0.1	0.4 ± 0.08	0.7 ± 0.02

Balb/c mice were challenged on day 0, 7, and 14 with PBS, aprotinin-treated PBS (PBS-Ap), GC frass (40 μg/40 μl) or aprotinin-treated GC frass (frass-Ap). On day 17, BAL fluid was harvested and differential cell counts performed. These data represent 6 mice per group and are expressed as mean ± SEM cell number ×10^4^. There were no statistical differences between PBS-Ap and PBS, nor were there differences between GC frass-Ap and GC frass. Statistical significance between GC frass and PBS treatments were determined by ANOVA (mac p < 0.001; eos p = 0.002; neut p < 0.001; lymph p < 0.001).

### GC frass induced airway inflammation and hyperresponsiveness in C57Bl/6 mice

**Figure 6:**
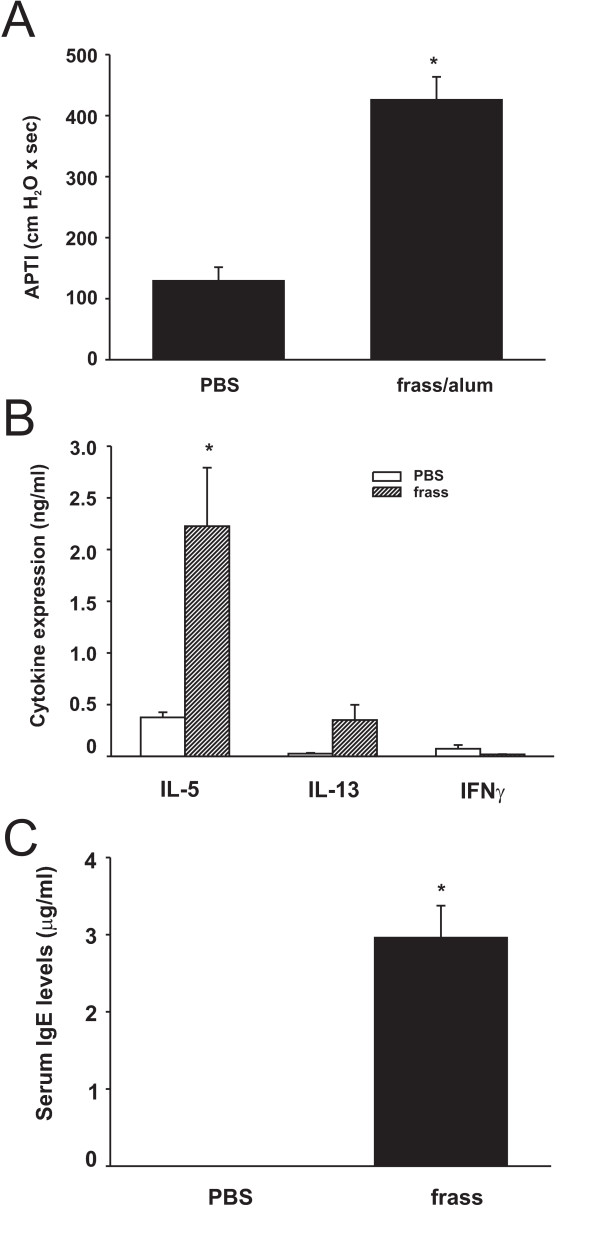
GC frass-induced experimental allergic asthma in C57Bl/6 mice. C57Bl/6 mice were sensitized on day 0 and 7 with an intraperitoneal injection of 100 ug/ml PBS or GC frass with alum. On days 14 and 19, an intratracheal inhalation was performed using PBS (40 μl) or GC frass (40 μg/40 ml). On day 22, mice were anesthetized acetylcholine was injected after establishment of a stable airway pressure. A. AHR was measured as airway pressure time index (APTI) in cm-H_2_O × sec ^-1 ^(* p = 0.016). B. Lungs from the mice were excised, and cells dissociated and maintained in a single suspension culture for 3 days in the presence of Con A (10 μg/ml). Supernatants were removed and ELISAs were run for IL-5, IL-13 and IFNγ. These data are represented as cytokine (listed on the x-axis) in ng/ml from PBS or GC frass treated mice (*p = 0.012). C. Serum IgE levels were analyzed by ELISA (*p < 0.001). In all cases the data are expressed as mean ± SEM and represent 6–7 mice per group and statistical significance determined by ANOVA.

**Figure 7:**
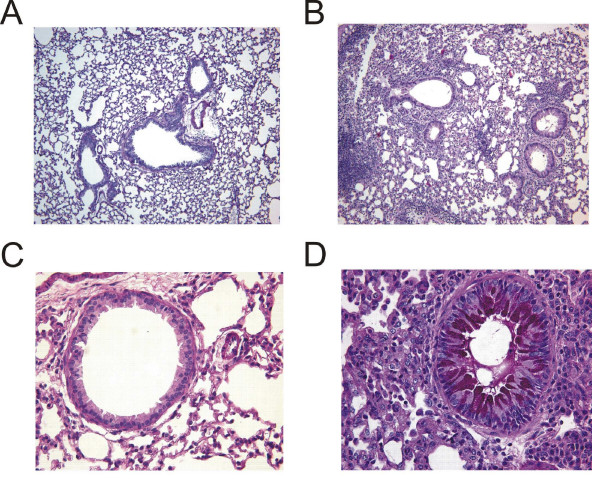
Histological assessment of lung sections from PBS or GC frass exposed C57Bl/6 mice. Haematoxylin and eosin (H&E) staining of sectioned lungs from PBS (A) and GC frass (B) treated C57Bl/6 mice. Periodic Acid Schiff (PAS) staining of sectioned lungs from PBS (C) and GC frass (D) treated C57Bl/6 mice. Representative slides are shown of sections from 6–7 mice per group.

Next we asked if GC frass was able to induce allergic asthma in C57Bl/6 mice (Figure 1B). Using this protocol, we were able to establish airway hyperresponsiveness to acetylcholine (Figure 6A). An increase in TH2 cytokine production and a decrease in TH1 cytokine production were also detected, although only the increase in IL-5 was statistically significant (Figure 6B). Serum IgE levels were significantly increased (Figure 6C). GC frass treatment also resulted in increased eosinophils, neutrophils, macrophages, lymphocytes and epithelial cells in the BAL fluid (Table 3). Histological examination of the lung following sensitization and challenge of GC frass compared to PBS showed dense perivascular and peribronchiolar infiltrates (Figure 7 A+B) and abundant mucin in epithelial cells (Figure 7 C+D). 50 ± 2% of the GC frass challenged airways were positive for mucin staining, compared to 8 ± 2% of the PBS challenged control mice. These data demonstrate that while C57Bl/6 mice unable to be sensitized to GC frass via intratracheal inhalation; they were susceptible to aggressive sensitization and challenge with GC frass, suggesting a difference in the airways between the C57Bl/6 and Balb/c mice.

**Table 3:** Differential cell count in BAL fluid of C57Bl/6 mice

	**Mac**	**Epi**	**Eos**	**Neut**	**Lymph**
PBS/alum	0.9 ± 0.07	0.9 ± 0.07	0	0.01 ± 0.003	0.02 ± 0.008
frass/alum	33.2 ± 2.1	6.8 ± 1.7	25.1 ± 6.1	8.1 ± 1.7	18.3 ± 3.7
p value	<0.001	0.019	0.008	0.004	0.003

C57Bl/6 mice were given intraperitoneal injections of PBS with alum (PBS/alum) or GC frass with alum (frass/alum) on day 0 and 7. Intratracheal inhalations of PBS or GC frass were performed on days 14 and 19. On day 22, BAL fluid was harvested and differential cell counts performed. These data represent 7 mice per group and are expressed as mean ± SEM cell number ×10^4^. Statistical significance between GC frass and PBS treatments were determined by ANOVA.

### Active serine proteases do not mediate airway inflammation and airway hyperresponsiveness in C57Bl/6 mice

Using the same sensitization and challenge protocol for C57Bl/6 mice (Figure 1B), we investigated the role of GC frass associated proteases by using GC frass pretreated with aprotinin. There was no effect of removal of GC frass proteases on airway hyperresponsiveness to acetylcholine, TH2 cytokine production, or serum IgE levels (data not shown). There was a significant inhibition of IFNγ production by removing the proteases from GC frass (data not shown). In addition, there was no significant difference between PBS bound to alum and aprotinin-treated PBS bound to alum for TH2 cytokine production, or serum IgE levels (data not shown). There was an increase in airway hyperresponsiveness to acetylcholine in the aprotinin-treated PBS compared to PBS bound to alum, but this increase was not statistically significant. These data demonstrate that GC frass-derived proteases elicit a direct effect on the airways to augment allergen-induced airway inflammation and hyperresponsiveness.

## Discussion

Using a method which reflects the natural exposure to environmental allergens, inhalation of GC frass induced allergic asthma as determined by increased TH2 cytokines in the BAL fluid, increased serum IgE levels, increased responsiveness to acetylcholine challenge, increased cellular infiltration into the airways and increased mucin production in Balb/c mice. The same inhalation protocol resulted in increased TH2 cytokines in C57Bl/6 mice, with the other parameters not being affected. C57Bl/6 mice were susceptible to sensitization and challenge with GC frass; however this required an aggressive sensitization and challenge protocol. The difference in allergen challenge suggests an inherent difference in the airways between these mice. In the Balb/c mice, which were susceptible to sensitization via intratracheal inhalation, we found that active serine proteases in GC frass played a role in regulating airway hyperresponsiveness to acetylcholine and mucin production. Removal of proteases from GC frass had no effect on the C57Bl/6 mice which required sensitization via intraperitoneal injection. Together these data show that in mice susceptible to sensitization by inhalation, GC frass related proteases play a role in augmenting the allergic asthma phenotype and suggests functional differences in the airways of the strains of mice tested in this study.

While both mouse strains were able to induce allergic experimental asthma following sensitization and challenge with GC frass, the mice differed in their susceptibility to GC frass. C57Bl/6 mice have a tendency towards TH1 and consistently produce high levels of IFNγ [23]. Balb/c mice on the other hand, show a tendency towards TH2 cytokine expression. This could be one explanation why C57Bl/6 mice were unable to be sensitized via inhalation challenge, and the use of the adjuvant alum, which promotes a TH2 response, was required. Consequently, we noted differences in magnitude of TH2 cytokine levels between the mice, with Balb/c mice having higher levels both at baseline and following stimulation. However, using the more aggressive sensitization and challenge protocols for the C57Bl/6 mice, similar increases in airway responsiveness to acetylcholine, serum IgE levels, and mucin production were detected. It is important to note that while allergic asthma could be induced in C57Bl/6 mice, it was not by a natural exposure to GC frass. Together these data suggest that functional differences in the airways of Balb/c and C57Bl/6 mice could lead to differences in airway susceptibility to allergen exposure.

The airway epithelium is the first contact between the lung and aeroallergens, viruses and irritants, and as such, the airway epithelium needs to respond appropriately. In response to these stimuli, airway epithelial cells produce inflammatory chemokines. That the airway epithelium can respond to allergen treatment has been shown in a number of studies. The house dust mite cysteine protease Der p 1 caused disruption of intracellular tight junctions, detachment of lung epithelial cells, and epithelial release of cytokines *in vitro *[24,25]. We have previously reported that GC frass contains active serine proteases which modulate cytokine expression via the activation of protease activated receptor (PAR)-2 in human bronchial epithelial cells *in vitro *[26]. PARs are a family of transmembrane spanning receptors that are activated upon cleavage by a variety of extracellular proteases. The role of PAR-2 in allergic respiratory disease has been documented but is still controversial. Since many of the allergens have been shown to contain active proteases, this may be a mechanism by which an allergen regulates the airway epithelium.

Other studies have investigated the role of proteases in modulating the experimental asthma phenotype in mice. In the highly responsive A/J mouse strain, proteolytic activity in the house dust mite allergen Der p 1 was shown to induce sensitization toward IgE responses by a cysteine protease-dependent mechanism [27]. Der p 1 has also been shown to induce cellular infiltration into the lungs in a protease-dependent manner in Balb/c mice [28]. Interestingly, both of the abovementioned models immunized mice with allergen bound to alum by intraperitoneal injection. Further work will shed additional light on the role of aeroallergen-derived proteases in the development of experimental asthma phenotype in mice.

A crucial step in mediating a T cell immune response is the uptake, processing and presentation of antigen by antigen presenting cells. It is possible that genetic differences in airway epithelial cells between the murine strains could lead to differences in allergen uptake and processing. The epithelium produces a number of chemokines which regulate cellular recruitment, and as such the epithelium could alter the immune response to allergens. Differences in airway epithelial biology could dampen the damaging effects of proteases inherent in allergens, or from the release of oxygen radicals, proteases and soluble mediators of inflammation from neutrophils. This could be accomplished by the synthesis of variable levels of endogenously expressed proteins such as alpha-1 antitrypsin or secretory leukoprotease inhibitor, both of which protect tissue against the destructive action of neutrophil elastase at the site of inflammation. Our data suggests that the airway epithelium plays an important role in the sensitization of mice to allergen, as exemplified by the different susceptibility of the Balb/c and C57Bl/6 mouse strains to induce experimental asthma.

In susceptible humans and animals, allergens induce TH2 driven production of IgE, airways hyperresponsiveness and peribronchial inflammation. But the question remains, what makes some humans susceptible? Our data show that lung susceptibility to allergen is different from other routes of sensitization, i.e. active proteases play an important role in the sensitization process in the inhalation model used for Balb/c mice. Sensitization via intraperitoneal injection of allergen with adjuvant also induces experimental asthma, but in a fashion independent of active proteases. The sensitization protocol for Balb/c mice mimics the natural route of allergen sensitization, i.e. inhalation of the allergen. In the allergen inhalation model of asthma we present, allergen-derived proteases play an important role in mediating allergic susceptibility. It will be of considerable interest to determine the role of active proteases in modulating human asthma.

## Abbreviations

APTI; airway pressure time index

BAL; bronchoalveolar lavage

CR; cockroach

Frass; feces

GC; German cockroach

IL; interleukin

OVA; ovalbumin

## Competing interests

The author(s) declare that they have no competing interests.

## Authors' contributions

KP designed and performed the experiments and drafted the manuscript. KML performed the immunoassays and analysis of the slides. NH performed the animal work. MWK participated in the design of the study and helped to draft the manuscript. All authors read and approved the final manuscript.
